# Workload Assessment of Technical Staff in a Clinical Laboratory in South India.

**DOI:** 10.12688/f1000research.152755.2

**Published:** 2025-10-30

**Authors:** Shalet Thomas, Somu Ga, Sushma Belurkar, Tarushree Bari, Asha Patil

**Affiliations:** 1 Medical Laboratory Technology, Manipal College of Health Professions, Manipal Academy of Higher Education, Manipal, Karnataka, India; 2 Department of Hospital Administration, Kasturba Medical College, Manipal Academy of Higher Education, Manipal, Karnataka, India; 3 Department of Pathology, Kasturba Medical College, Manipal Academy of Higher Education, Manipal, Karnataka, India; 4 Directorate of Online Education, Manipal Academy of Higher Education, Manipal, Karnataka, India; 5 Medical Laboratory Technology, Manipal College of Health Professions, Manipal Academy of Higher Education, Manipal, Karnataka, India

**Keywords:** Clinical Laboratory; Human resources management; Healthcare ; Technician; Standard workload; Staff requirement; Work-force; Workload Indicators for Staffing Need (WISN)

## Abstract

**Background:**

Clinical laboratories play a significant role in healthcare, providing tests for diagnosis and treatment monitoring. Having skilled and adequate staffing is crucial for clinical laboratories to operate efficiently, manage their workload effectively, and deliver timely results. Despite being critical for healthcare delivery, ensuring adequate staffing levels in clinical laboratories remains a complex challenge for healthcare providers worldwide due to resource constraints and workforce shortages.

**Methods:**

This study evaluates the workload in clinical laboratory staff using Workload Indicators of Staffing Need (WISN) method, focusing on workload, work pressure, and staffing requirements to optimize staffing levels and ensure an efficient clinical laboratory environment. Annual clinical laboratory data from June 2021 to May 2023 were collected to calculate the average number of days (234) worked annually, percentage of workload, distribution of activity time measurement units, and the ratio of needed, surplus, and existing staff (n=33, complete enumeration) from the laboratory registers and hospital statistics. WISN software was used to calculate staffing requirements, workload pressure, and WISN ratio. The Shapiro–Wilk test assessed normality and the Kruskal–Wallis test evaluated differences across clinical laboratory sections.

**Result:**

A total of 33 technical staff employed, performed approximately 3.7 million tests across hematology and clinical pathology during the study period, WISN ratios ranged from 0.2 to 0.7, indicating the existing 33 staff members face a high workload burden. The calculated ideal staffing level was 46.33 personnel, indicating a shortfall of 13.3 staff (28.3%). A p < 0.01 confirmed a significant disparity in workload distribution.

**Conclusion:**

The results highlight the importance of optimizing staffing levels in clinical laboratories to ensure quality service delivery. The WISN methodology can be a useful tool in healthcare facilities for making evidence-based staff allocation, maximizing the utilization of employee skill sets, and establishing standard staffing benchmarks tailored to the needs of clinical laboratories.

## Introduction

Efficient healthcare relies heavily on the valuable contributions of clinical laboratory personnel. These skilled professionals help diagnose illnesses and effectively monitor treatments. Their expertise is genuinely indispensable for making informed decisions and delivering top-notch care. The effective use of clinical laboratory services improves doctors’ capacity to use the least resources possible while reducing total healthcare costs by helping them make evidence-based diagnostic and treatment decisions for their patients. Clinical laboratory services are the least intrusive and economical source of objective health information for preventing and diagnosing diseases, enhancing patient outcomes, guaranteeing patient safety, and carrying out crucial public health monitoring tasks ( Swanson et al., 2018). A crucial component of health care is clinical laboratory services. As a result, adequate staff capacity is required to ensure patient satisfaction and improve patient and clinical staff outcomes ( Tripković et al., 2020). Human resources for health are essential for providing healthcare services to the population ( Nandan & Agarwal, 2012).

The World Health Organization (WHO) developed Workload Indicators of Staffing Need (WISN), a human resource planning and management tool that gives health managers a mechanism to assess and determine the ideal staffing levels in healthcare facilities ( Shipp & WHO, 1998; Pandey et al., 2013). The WISN method is a tool for human resource management that calculates the number of health workers needed to meet the demands of a specific healthcare institution and evaluates the workload pressure experienced by those workers. This method has several advantages, including being easy to use and applicable in any healthcare setup and other institutions that may not be medical (WISN: user’s Manual, second edition-2023, Asres & Gessesse, 2024).

Health planners and policymakers must ensure that workers with the necessary skills are available appropriately. Policymakers should contribute critical insights to developing guidelines for healthcare facilities’ staffing standards in the context of resource constraints ( Stankovic & Santric Milicevic, 2022). The acknowledgement of the importance of clinical laboratory for patient welfare remains the most valuable attribute for any healthcare worker, despite the ongoing problems they encounter. Even if the public and patients are unaware of its existence, clinical laboratories’ primary purpose is to provide superior clinical laboratory diagnostic tests ( Bayot et al., 2023). The need for more human resources to match the needs of their communities is posing a problem for healthcare management everywhere. The significance of health professionals’ accessibility in attaining universal health coverage and sustainable development objectives has been emphasized by the global health human resources strategy, Workforce 2030 ( Gupta et al., 2023).

Diagnosis and treatment these days are primarily driven by evidence-based; the hematology and Clinical Pathology Laboratory is one of the most supporting medical units, and this functional unit plays a significant role in supporting hospital operations by testing the cause of the disease. Scarce research is available in the staffing and utilization of clinical laboratory staff. This exploration aids in assessing the effective use of technical staff in the context of workload, work pressure, and staff requirements in the clinical laboratory.

## Methods

The study was initiated after obtaining approval from the Institutional Ethics Committee, Kasturba Medical College (KMC) and Kasturba Hospital (KH) (IEC2-345/2023) (approved on 22 July 2023), Institutional Research Committee (IRC- MCHP-Mpl/IRC/PG/2023/150, 18 April 2023). WISN software is accessible upon registering with the WHO, making it an efficient and valuable tool for our research endeavors.

As human participants not included, no consent required for the performed study. However, data was collected with permission from clinical laboratory in charge and head of the hospital while obtaining IRC and IEC approval.

The study was conducted in the clinical laboratory of a tertiary care hospital., operating 24/7 with a staff of 33 technicians (complete enumeration). The data on workload, distribution of activity time, staff Available Work Time (AWT), time spent by staff for the supportive activities (such as training, meetings, sample pre examination procedures and equipment maintenance) were collected over a period of two years, from June 2021 to May 2023 from clinical laboratory registers and hospital statistics.

### Scope of the study

This study may help guide decisions during the planning stage of enhancing the capacity and calibre of clinical laboratories. Both small and large workloads can negatively impact employees’ skills and workplace attitudes. Hence, clinical laboratories must have competent staff to handle the workload. In emergency conditions, the number of clinical laboratory tests that must be performed daily grows exponentially, and the time needed to complete core operations doubles. As a result, more workers are needed. Additionally, it issues a warning that workforce management and planning must be predicated on several circumstances of the demand for clinical laboratory services and capabilities, necessitating the professional engagement of HRM planners.

### Data collection

Data was obtained from the clinical laboratory registers which included details regarding work hours, daily tasks performed by staff, and the typical workload encountered and other activities to achieve the task Other Quantitative data were procured from the information technology department which encompassed service of the staff, leave taken, number of holidays per year, and the total number of hematology and clinical pathology tests conducted.

### Data analysis and interpretation

Annual hospital statistics were collected, and the WISN methods were applied to the clinical laboratory, which has several analytical sections, autoanalyzers and partial manual tests. All data collected from the clinical laboratory records and annual service statistics were analyzed using WISN software ( WISN Eng Software manual for Web - apps.who.int. - User’s Manual, 2 nd ed. 2023) World Health Organization (2023). WISN: workload indicators of staffing need: user’s manual, 2nd ed. World Health Organization. Licensed under the Creative Commons Attribution-NonCommercial-ShareAlike 3.0 IGO (CC BY-NC-SA 3.0 IGO) license. https://www.who.int/about/policies/publishing/copyright ( Copyright (who.int), https://www.who.int/publications/i/item/9789240070066).

### WISN (WHO) procedure

The study followed the WHO/WISN manual instructions to estimate the technical staff requirement, including the following steps.

**Determining the priorities of staffing** – This is the decision to prioritize which category of staff must work in which section, e.g. The technical staff in the Flowcytometry section. **Estimating Available Working Time** (AWT) is the total working hours available for each clinical laboratory technical staff in a year, and it is the technologists/technicians who are available in one year to do their work, considering contractually and legally authorized and unauthorized absences.

**Workload Components for clinical laboratory staff** include main activities, additional categorical activities, which all staff members perform, and additional individual activities, which are performed only by specific (not all) staff members.

**Setting Activity Standards** - An activity standard is how long it takes a trained, skilled, and motivated individual to complete an activity to a clinical laboratory standard.

There are two kinds of activity standards: service standards for health service activities, which determining the time required for each activity performed by the staff and allowance standards, which serve as activity standards for supporting and supplemental activities. Allowance standards are calculated separately for support and additional activities, as Individual Allowance Standards [IAS], and Category Allowance Standards [CAS]. The percentage of AWT spent on each support activity determines CAS (given in percentage). The number of clinical laboratory staff members is multiplied by the time needed for each extra activity in a year to calculate IAS (stated in time units).

**Establishment of Standard Workloads** - determines the amount of work a technical staff can complete annually across all fundamental clinical laboratory tasks.

Standard workload = Available Working Time (AWT) in a year multiplied by the rate of work.

**Calculating Allowance Factors** - An allowance factor is calculated separately for support and additional activities. This refer to time adjustments for non-service activities (such as administrative duties, training, or meetings) and do not represent financial allowances or monetary compensation. Support activities - Category Allowance Factors (CAF). Additional activities - Individual Allowance Factors (IAF)

CAF = 1 / [1 - (Total CAS / 100)], where CAS is the percentage of time spent on support activities.

**Determine staff requirements** - staff required for clinical laboratory workload. Divide an annual clinical laboratory workload for each component by its standard workload. This will be the number of staff required for the clinical laboratory activity, and all the workload components will be added together, giving the total clinical laboratory staff required. Staff requirements = annual clinical laboratory workload for each component/standard workload

### Data analysis

*Determining the priorities of staffing and estimating available working time*

The staff 33 technical staff, were categorized as a laboratory technologist and in charge, laboratory technologist, senior technician, laboratory technician grade I, and laboratory technician grade II.

*The Available Working Time (AWT)*

This step calculates the staff’s actual working days for one year. Then, the days the staff may not work in a year are determined for various reasons.

*Calculating available Working Time (AWT)*

AWT=A–(B+C+D+E)

where:

• AWT is the total available working time
• A is the number of possible working days in a year
• B is the number of days off for public holidays in a year
• C is the number of days off for annual leave in a year
• D is the number of days off due to casual leave in a year
• E is the number of days off due to other leave in a year.

$$\begin{array}{l} \mathrm{AWT}=A–\left(B+C+D+E\right)\\ \mathrm{AWT}=312–\left(12+29+12+25\right)=312-78\\ \;=234\;\text{days}/\text{year} \end{array}$$

The working days are then converted to hours by multiplying AWT in working days by daily working hours. **F** is the number of working hours a day, i.e., 8.

$$\begin{array}{l} \mathrm{AWT}=\left[A–\left(B+C+D+E\right)\right]\times F\\ \mathrm{AWT}=234\times 8\\ \;=1872\;\text{hours}/\text{year}=112320\;\text{minutes}/\text{year} \end{array}$$

### Determining workload components and establishing standard workloads

The workload components are the activities of the technical staff’s daily schedule. Then, we set a standard workload based on the information obtained by the available working time and established a standard workload for each workload component in the clinical laboratory.

Standard workload = “AWT in a year divided by unit time” or “AWT in a year multiplied by a rate of working”. Activities per hour are a measure of the rate of working ( Table 1).

**Table 1.** Workload components, Standard workload, annual number of tests and staff requirements.

Activity name	No. of tests Per Year	Service Standard *	Standard Workload	Calculated staff Requirement
Complete Blood Count+ Retic +ESR	1404293	1	112320	12.5
QBC Malaria &Microfilaria, Malaria Smear	3787	30	3744	1.01
Special test in Blood and coagulation section **	8361	15-180	624-7488	2.07
Coagulation tests	82041	8	14040	5.8
Urine Analysis Complete	51091	5	22464	2.18
Fluid Analysis + CSF	3738	30	3,744.00	0.99
Staining of smears (bone marrow)	4800	30	3744	1.28
Phlebotomy	230319	10	11232	20.5

* The unit of reporting service standard is minutes/test except for phlebotomy (minutes/patients).

** Low number of tests with high service standards.

### Calculating allowance factors

Types of allowance standards: category allowance standards (CAS) and individual allowance standards (IAS), and an allowance factor is calculated separately for support and additional activities.

CAF=1/[1−(TotalCAS/100)]

In this study, clinical laboratory technician grade -II spends 126 minutes (equal to 2.1 hours) on activities supporting service activities (e.g., reagent preparation, QC analysis, startup and shutdown of analyzers, smear preparation, and receiving and segregation of samples, etc.).

The CAS % can be calculated as 2.1 hours multiplied by 234 (annual working days)/1872 (AWT) multiplied by 100

$$\begin{array}{l} =\left(491.4÷1872\right)\times 100=0.2625\times 100=26.25\;\text{or}\\ \mathrm{CAS}\%=\left(2.1\;\text{hours}÷8\;\text{working hours}\;\mathrm{per}\;\mathrm{day}\right)\times 100=26.25\\ \mathrm{CAF}=1÷\left(1-\text{TOTAL}\;\mathrm{CAS}÷100\right)\\ =1÷\left(1-0.2625÷100\right)\\ =1÷0.7375\\ =1.3559=1.36 \end{array}$$

The IAF calculation divides the annual total IAS by the AWT.

Here in this study clinical laboratory Technician grade II has an annual IAS of 286 hours, and AWT is 1872 hours, then

$$\begin{array}{c} \text{Individual Allowance Factors (IAF)}=\text{Annual IAS}/\mathrm{AWT}\\ =286/1872=0.15 \end{array}$$

### Standard allowance

The technical staff has an hour or 60-minute lunch break, a 15-minute tea break, and a standard allowance per week of 1.15 × 6 = 6.9 hours and allowance per year = 6.9 × 52 weeks = 358.8 hours/year. Standard allowance = average per allowance factor/Available working time = 358.8/1872 = 0.19 hrs/year ( Table 2).

**Table 2.** Summary of allowance factors and working time.

Parameter	Value	Explanation
Available Working Time (AWT)	1,872 hours/year	48 hours/week × 39 working weeks
Category Allowance Standard (CAS)	26.25%	Support activities (QC, reagent prep, startup/shutdown)
Category Allowance Factor (CAF)	1.36	1 ÷ (1 − 0.2625)
Individual Allowance Factor (IAF)	0.15	286 ÷ 1,872
Standard Allowance (per year)	0.19 hrs	Lunch/tea breaks and weekly allowances

### Estimation of staff requirements

The technical staff requirement is obtained by dividing the annual workload for each workload component by its standard workload and by adding all workload components together = the total staff required in the clinical laboratory (Workload Indicators of Staffing Need - User’s Manual, n.d. 2023).

For example: To perform the Complete Blood Count, the required staff = Total number of tests (annual workload)/standard workload = 1404293/112320 = 12.5.

### Statistical analysis

Along with WISN, the Shapiro-Wilk test was utilized to assess the normality of the data. A p-value below 0.01 indicated that the data originated from a non-normal distribution. Consequently, the Kruskal-Wallis test was conducted as a non-parametric alternative to ANOVA. A p-value less than 0.01 indicated a notable disparity in the demand for technical staff and workload across each section of the clinical laboratory.

## Results

### Staff and working hours

The study included all 33 technical staff working in the clinical laboratory. The staff ages ranged from 24 to 56 years, with experience levels varying from 5 to 36 years. Each technician works a standard 48-hour week (8 hours per day with a 1-hour 15-minute break), resulting in an annual available working time (AWT) of 1872 hours after accounting for non-working days.

### Clinical laboratory activities

Clinical laboratory technicians perform various tasks across the pre-analytical, analytical, and post-analytical phases. These include sample reception and registration, sample processing, running assays on analyzers, data entry, and report verification. Additionally, they assist with patient procedures, reagent preparation, quality control, and other support activities. The specific workload components for each technician are determined by their daily schedule and the types of tests performed.

### Workload and staff requirements

In two years (June 2021 to May 2023), 4593430 patients were registered, and approximately 3692642 tests were performed, including phlebotomy. On average, per year, 1558111 tests and 230319 phlebotomies were done. The WISN method was used to analyze staffing requirements based on available working time and activity standards. The WISN data provided workload standards in minutes per activity and served as the basis for calculating the standard annual workload for each component. We then followed the steps outlined in the WISN manual to assess workload pressure and staff needs. (Workload Indicators of Staffing Need - User’s Manual, n.d.2023) The difference and ratio generated by the WISN software will be further analyzed in the discussion section to understand staffing adequacy ( Table 1).

### Allowance factors

Supporting activities in the clinical laboratory consumed 15.63% to 31.73% of annual working time. The category allowance factor (CAF) indicated that 1.19% to 1.46% of technical staff time should be dedicated to both primary and supporting activities. Individual allowance factors (IAF) ranged from 0.03 to 0.34, accounting for additional tasks performed by specific staff members.

The WISN analysis assessed staffing adequacy through two key metrics:

1. Difference: The difference between the current and required number of staff indicates understaffing or overstaffing.
2. WISN Ratio: This ratio reflects workload pressure, with a value of 1 indicating balanced staffing, >1 suggesting overstaffing, and <1 indicating understaffing.

### Available working time (AWT)

The available working time (AWT) for clinical laboratory technicians was determined by subtracting the total number of non-working days (78 days excluding Sundays) from the total possible working days (312 days). There are 52 weeks a year, with 39 working weeks and 13 non-working weeks. Each clinical laboratory technician works 48 hours per week, which translates to 8 hours daily, including a 1-hour fifteen-minute break. Therefore, the total annual working hours amount to 1872.

In the clinical laboratory, significant levels of work overload and workload pressure were experienced by the section where the WISN ratio of 0.2 to 0.7 and the WISN ratio from 1 to 1.6 revealed that sections had no workload pressure. The WISN tool states that the workload increases with a lower ratio ( *Workload Indicators of Staffing Need - User’s Manual*, n.d.). Based on the Staff category WISN Ratio, each category of technical staff exhibits an overload, with WISN ratios ranging from 0.03 to 0.16.

Upon completion of a workload study, the technical staff ratio assesses the existing number of technical staff against the actual requirement for technical personnel. The data analysis revealed a disparity between the number of technical workers needed and those currently available in the clinical laboratory ( Table 3).

**Table 3.** Actual staff and WISN-based staffing estimates with workload pressure in the clinical laboratory.

Section	Actual staff	WISN-based required staff	Difference	Staff status	WISN ratio *	Workload pressure
Phlebotomy Section	14	20.5	−6.5	Shortage	0.7	Moderate
Blood Section & Analyzer	4	12.5	−8.5	Shortage	0.3	High
Bone Marrow Staining	2	1.28	+0.72	Surplus	1.6	Low
Flow Cytometry	1	2.07	−1.1	Shortage	0.5	High
Fluids & QBC	1	1.0	0	Balanced	1.0	Normal
Coagulation	1	5.8	−4.8	Shortage	0.2	High
Urine Analysis	1	2.18	−1.2	Shortage	0.5	High

* WISN Ratio = Actual/Required Staff; Ratio < 1 = Shortage, Ratio > 1 = Surplus.

## Discussion

The clinical laboratory technical staff’s efficiency is critical for ensuring high-quality patient care and efficient diagnostics ( Ebubekir et al., 2017). In this study, the WISN methodology was applied to objectively assess staffing adequacy in a tertiary care clinical laboratory. The WISN-based approach provides a data-driven measure of workload distribution rather than relying on subjective perceptions, allowing rational redistribution of staff resources. Considering the rapidly evolving healthcare landscape, no other medical specialities alone can fully meet the complex needs of patients. As such, clinical laboratory technicians should play a critical role in driving clinical effectiveness and improving patient care ( Ferraro et al., 2016). Their accurate diagnoses and efficient treatment support are integral to ensuring optimal patient outcomes ( Cortelyou-Ward et al., 2011).

A study highlights the critical role of human resource planning and management in the healthcare sector, particularly within clinical laboratory environments. Research involved a large group of health laboratory workers, medical laboratory technicians, and medical biochemists, illustrating the diverse personnel involved in such services ( Stankovic & Santric Milicevic 2022).

In a District Hospital of Madhya Pradesh, the clinical laboratory technicians were constantly under pressure to match the existing workload due to shortage of 10 manpower. Hence, it indicates an urgent need for improved human resource management in the healthcare facility ( Pandey et al., 2013). In the current study, the clinical laboratory conducts a significant number of tests annually, indicating a substantial workload. Despite this high volume, the clinical laboratory efficiently manages its operations with a relatively small team of 33 technical staff. This emphasizes the crucial role of efficient resource allocation and management strategies within clinical laboratory settings to ensure optimal productivity and service quality.

The field of medical laboratory science is overwhelmingly female, with women comprising 88.99% of medical laboratory technicians and 81.8% of medical biochemists, with an average age of 50 ( Stankovic & Santric Milicevic 2022). This trend is further reflected in the current study, where 94% of the technical staff are women. The age range (24 to 56 years) of these technicians highlights their extensive experience, varying from recent graduates to seasoned professionals with over 30 years of service. This diversity highlights the strong expertise within the team.

A scarcity of technical staff, an ageing workforce, and ticking demographic time bombs of pension-related issues all threaten to compound the situation. The impact of these challenges is evident in delayed diagnoses and errors, directly impacting patient care quality ( Cortelyou-Ward et al., 2011; Yenice, 2020; Strain & Sullivan, 2019). To combat these challenges head-on, it is crucial to diversify the technical staff by appointing individuals across different age groups and experience levels ( Green et al., 2002). In the current study, the diverse team of technical staff serves as the backbone for sustaining top-notch service quality and keeping the clinical laboratory up to speed amidst the fast-paced evolution of cutting-edge technologies.

The midwives at Asrade Zewude Memorial Hospital worked five working days with an actual working time of 1030 hours annually. 58.4% of hours were spent on health service activity, additional activities, 36.6% hours, and supporting activities, 5% hours ( Asres, 2023). The time that laboratory personnel spent on main activities was 70%. Per core activities took 0.25 to 180 minutes ( Tripković et al., 2020). In the current study, the total available working time was 112320 minutes/year and 1872 hours/year, and the technical staff spent 68 to 84% of the time on main activities and 1.19 to 1.46% on supporting activities, individual allowance factor (IAF) for additional activities only to specific staff members 0.03 to 0.34. The inpatient medical record data update personnel were granted an allowance of 286 hours/year and a standard allowance of 0.17 hours per year ( Prihadi et al., 2021). In the current study, the allowance for technical staff is 358.8 hours/per year, and the standard allowance is 0.19 hours/per year.

In a study in the RSU Anutapura Palu clinical laboratory, reveals that the productive time used for activities was 88.6%, and the working hours was 114,240 minutes/year. The workload standard was 5817.32 annually, and the allowance was 0.4% annually. Employee in clinical laboratory unit distribution has been successful; tasks and activities divided into productive, unproductive, and personal pursuits. Each employee has a 0.4 annual leeway factor, almost 40%. The clinical laboratory has a deficit of 8 technicians. Qualification and competence are important for manpower planning. Understanding the optimal staffing levels can help hospital management make informed decisions regarding recruitment, training, and retention of staff members, ensuring that the workforce is adequate and competent to handle the workload ( Napirah & Sulistiani, 2015).

The WISN difference shows a 20% and 14% Full-Time Equivalent (FTE) shortfall for medical biochemist and medical laboratory technician, respectively, with inconsistent workload pressure/WISN ratio of 0.4 to 1.0 ( Stankovic & Santric Milicevic 2022). In a similar study by Tripković et al., there was a shortage of 22.22% laboratory analysts and 20.63% laboratory technicians and moderate to high workload pressure ( Tripković et al., 2020). In the current study, the variation in WISN difference across sections was striking, ranging from 4.9% to 37.8%. Comparable findings have been observed in WISN studies from Indonesia, Kenya, and India, where WISN ratios below 1.0 reflected staff shortages and workload intensity (WHO, 2010; Pandey et al., 2013; Tripković et al., 2020). The current study’s WISN ratios (0.2–0.7 in high-volume sections) align with these reports, confirming that clinical laboratories are among the most workload-sensitive departments in healthcare settings. However, two sections demonstrated balanced staffing, indicating effective workforce allocation.

There was a significant difference between the number of employees currently employed and the number calculated by WISN. 25% of the employees were accessible to meet their needs, and 75% were shortfalls ( Shivam et al., 2014). Three dialysis technicians needed to give patient care, and the hospital hired two of them ( Gupta et al., 2023). There was a severe shortage in the Nurse/Midwife category, and there were just nine staff available where 45 were needed ( Oaiya et al., 2022). In the current study, the WISN calculation has determined that the clinical laboratory needs a total of 46.33 staff to handle the influx of samples and effectively manage the workload. However, with only 33 (71.7%) technical staff currently employed, there is a notable deficit of 13.33 additional staff. This represents a significant 28.3% increase required to ensure the smooth functioning of the clinical laboratory and to manage the workload adequately.

A moderate to high workload is shown on the WISN ratio, which ranges from 0.86 to 0.50 for clinical laboratory workers ( Tripković et al., 2020). A staff difference of 0 and a WISN ratio of 1, indicating that there were just enough personnel on hand to provide the facility’s services by national professional standards. Therefore, maintaining the services was the only necessary activity ( Dimiri et al., 2022). In a few sections of the current study, the difference in technical staff runs from 1.1 to 8.5, showing a technical staff deficit. The WISN ratio ranges from 1 to 1.6, suggesting minimal workload pressure, and from 0.2 to 0.7, exposing moderate to high workload pressure. When a diagnosis is delayed or a mistake occurs, it lowers the quality and optimal treatment that the patient receives, which is when the effects of insufficient clinical laboratory workers become evident ( Cortelyou-Ward et al., 2011).

Despite encountering a heavier workload, the present study indicates that the reporting turnaround time was commendable. This was achieved through the redistribution of tasks among sections and the delegation of selected activities to graduates and students under supervision. This approach not only benefited patient care but also provided valuable training opportunities for future clinical laboratory professionals. Considering the significant workload, this strategy played a pivotal role in reducing the turnaround time. It provided students with a unique opportunity to enhance their skills and become proficient clinical laboratory technicians equipped with the education and experience necessary for success in their field of work. From a management perspective, these findings highlight the importance of integrating WISN data into strategic human resource planning and hospital budgeting. While the WISN method objectively determines staffing adequacy, future research should integrate cost–benefit and economic evaluations to assess feasibility, incentive mechanisms, and long-term workforce sustainability.

The WISN approach serves as a valuable tool for HRM planners, assisting in the determination of whether there is an excess or deficit of human resources due to work-related stress. The establishment of suitable HRM policies requires consideration of current labour market analysis based on reliable data ( Najafpour et al., 2023), and can help improve the distribution and equity of health workers within regions or similar healthcare facilities nationwide ( Satish Kumar et al., 2016). Although the current study shows that workload pressure among technical staff is moderate to high pressure and there is a technical staff shortage, the management of the hospital has considered the clinical laboratory and updated the number of technical staff. Overall, workload-based staffing analysis using the WISN method can serve as a foundation for evidence-based workforce planning, improving staff balance, service quality, and preventing burnout in high-volume clinical laboratories.

Using non-parametric Kruskal–Wallis one-way ANOVA (analysis of variance), facility-specific raw (unrounded) WISN values for all cadres were evaluated for a cross-state differences to investigate data heterogeneity. Since the count data produced by the WISN rounding technique caused problems with saturation raw numbers, a better fit was used in the ANOVA model. A non-parametric test was selected due to the data’s apparent skewness ( Nair et al., 2022). In the current study, the Shapiro-Wilk test was utilized to assess the normality of the data. A p-value <0.01 indicated that the data originated from a non-normal distribution. Consequently, a non-parametric Kruskal-Wallis test was conducted. A p-value < 0.01 indicated a notable disparity in the demand for technical staff and workload across each section of the clinical laboratory.

## Conclusion

The dedicated technological tool provided by the WHO for the WISN Software process facilitated the measurement of workload and the evaluation of technical staff utilization and needs. Based on the WISN, this study’s computation reveals a significant discrepancy between the actual workforce 33 and the WISN-calculated demand 46.33. The WISN results proved that staffing minimum and maximum standards and technical staff are optimally utilized in clinical laboratories. The WISN ratio is < 1, and the required number of clinical laboratory staff is 13.33, indicating the workload pressure. This study focuses on staffing needs in modern clinical laboratories, which are increasingly automated compared to traditional facilities. This unique approach can inform policies for developing workforce requirements in future clinical laboratories and healthcare settings.

## Limitations

The calculations for staffing requirements are based on data from previous years and focused on a single clinical laboratory of a single tertiary care hospital of South India, which may affect, the generalizability of the findings. However, adjustments may be required to account for the current year’s workload and staffing needs. The data were collected during the COVID-19 pandemic, which may have influenced staffing availability and workload patterns (e.g., absenteeism, emergency testing demands). This context may partly explain the higher workload pressure observed.

The WISN tool is helpful, but it has limitations. It may not fully consider facilities that operate all day and night or cases where different staff groups do similar tasks. Also, incorrect workload data could make the system suggest too few staff members. Despite its limitations, the WISN tool remains a valuable resource for healthcare staffing decisions. It works best in combination with other approaches.

## Future scope of the study

Financial allowances and compensation are important considerations in managing overworked staff, however, the WISN tool does not directly assess these aspects. Future studies incorporating economic assessments and cost benefits analysis alongside WISN analysis would provide a more comprehensive understanding of the workforce planning and staff motivation.

## Ethics and consent

The study was initiated after obtaining approval from the Institutional Ethics Committee, Kasturba Medical College (KMC) and Kasturba Hospital (KH) (IEC2-345/2023) (approved on 22 July 2023). The WISN software is accessible upon registering with the WHO, making it an efficient and valuable tool for our research endeavors. As human participants were not included, no consent was required for the performed study. However, data was collected with permission from clinical laboratory in charge and head of the hospital while obtaining IEC approval.

## Data Availability

Figshare: Unveiling efficiency: A research inquiry into technical staff utilization in South Indian clinical laboratory using workload indicators staffing need (WSN) analysis, https://figshare.com/articles/dataset/wisn_data_docx/27160689?file=49594527( Asha Patil et al., 2024).
1. WISN_Data file. Data are available under the terms of the Creative Commons Attribution 4.0 International license (CC-BY 4.0)
