# Supplementary material for: Workload Assessment of Technical Staff in a Clinical Laboratory in South India
Source: F1000Res. 2026 Apr 27;13:1319. Originally published 2024 Nov 5. [Version 4] doi: 10.12688/f1000research.152755.4 (PMC13198695; doi:10.12688/f1000research.152755.4)
Supplement: Peer review history

# Reviewer report: Reviewer response for version 4

**DOI:** 10.5256/f1000research.193851.r482469

**Published:** 2026-05-23

**Authors:** Ellen Kitson-Reynolds

**Affiliations:** 1 University of Southampton, Southampton, England, UK

Thank you for giving me the opportunity to review this paper. It is an interesting and valuable topic area and research.

 Abstract – concise overview of the paper

 Keywords - clear for search strategies

 Introduction – You present a clear contextualisation to the study. I suggest that the final paragraph in this section needs refining as suggested: - ‘Diagnosis and treatment these days are primarily driven by an evidence-base. The hematology and Clinical Pathology Laboratory is one of the most supporting medical units, and this functional unit plays a significant role in supporting hospital operations by testing the cause of the disease. Scarce research is available in the staffing and utilization of clinical laboratory staff. This exploration aids in assessing the effective use of technical staff in the context of workload, work pressure, and staff requirements in the clinical laboratory’

 Methods – Please add amend this sentence as suggested here: As human participants are not included, no consent was required for the performed study. However, data were collected with permission from clinical laboratory in charge and head of the hospital while obtaining IRC and IEC approval.

 Scope of the study – a clear presentation

 Data collection – Data are plural therefore I have amended the first sentence as suggested here and suggested a rephrasing of the remainder of the section: ‘Data were obtained from the clinical laboratory registers. These included details regarding work hours, daily tasks performed by staff, the typical workload encountered and other activities to achieve the task. Other quantitative data were procured from the information technology department. These encompassed service of the staff, leave taken, number of holidays per year, the total number of hematology and clinical pathology tests conducted.’

 Data analysis and interpretation – a clear section, thank you

 WISN (WHO) procedure - a clear section, thank you

 Data analysis – I am not a statistician; therefore, my comments may be limited in this section.  You have very clearly shown the step-by-step application to your working out of hours which is extremely helpful to the reader.

 Results – a clear section, thank you

 Discussion – This is a very interesting discussion. I wonder if it is appropriate to consider the sustainability of the cost for workforce considering a global financial and cost of living crisis. There is evidence of increasing workloads, but organisations don’t have increasing finances. Most organisations are having to do more for or, with less. Is this an issue moving forwards. It might be out of scope of your project, but it could be worthy to note. For example, extra workload + reduced staffing = burnout = less staff = poor services.

 Conclusion – concise

 Limitations - a clear section, thank you

Is the work clearly and accurately presented and does it cite the current literature?

Yes

If applicable, is the statistical analysis and its interpretation appropriate?

I cannot comment. A qualified statistician is required.

Are all the source data underlying the results available to ensure full reproducibility?

Yes

Is the study design appropriate and is the work technically sound?

Yes

Are the conclusions drawn adequately supported by the results?

Yes

Are sufficient details of methods and analysis provided to allow replication by others?

Yes

Reviewer Expertise:

Nursing and Midwifery

I confirm that I have read this submission and believe that I have an appropriate level of expertise to confirm that it is of an acceptable scientific standard.

# Reviewer report: Reviewer response for version 4

**DOI:** 10.5256/f1000research.193851.r479508

**Published:** 2026-04-28

**Authors:** Nasar Yousuf Alwahaibi

**Affiliations:** 1 Biomedical Science Department, College of Medicine and Health Sciences, Sultan Qaboos University, Muscat, Oman

Approved, no further comments

Is the work clearly and accurately presented and does it cite the current literature?

Partly

If applicable, is the statistical analysis and its interpretation appropriate?

Partly

Are all the source data underlying the results available to ensure full reproducibility?

Partly

Is the study design appropriate and is the work technically sound?

No

Are the conclusions drawn adequately supported by the results?

Partly

Are sufficient details of methods and analysis provided to allow replication by others?

No

Reviewer Expertise:

Histopathology

I confirm that I have read this submission and believe that I have an appropriate level of expertise to confirm that it is of an acceptable scientific standard.

# Reviewer report: Reviewer response for version 3

**DOI:** 10.5256/f1000research.191605.r441782

**Published:** 2025-12-30

**Authors:** Nasar Yousuf Alwahaibi

**Affiliations:** 1 Biomedical Science Department, College of Medicine and Health Sciences, Sultan Qaboos University, Muscat, Oman

Thank you.

 I recommend to add this small size number (33) as a limitation

Is the work clearly and accurately presented and does it cite the current literature?

Partly

If applicable, is the statistical analysis and its interpretation appropriate?

Partly

Are all the source data underlying the results available to ensure full reproducibility?

Partly

Is the study design appropriate and is the work technically sound?

No

Are the conclusions drawn adequately supported by the results?

Partly

Are sufficient details of methods and analysis provided to allow replication by others?

No

Reviewer Expertise:

Histopathology

I confirm that I have read this submission and believe that I have an appropriate level of expertise to confirm that it is of an acceptable scientific standard, however I have significant reservations, as outlined above.

# Response

**Published:** 2026-04-10

**Authors:** DR ASHA PATIL

**Affiliations:** Medical Laboratory Technology, Manipal Academy of Higher Education, Manipal, Karnataka, India

Thank you for highlighting the importance of acknowledging the workforce size as a limitation. We have added a concise statement to the *Limitations* section noting that the relatively small workforce size (n = 33) may limit the generalizability of the study findings.

# Reviewer report: Reviewer response for version 2

**DOI:** 10.5256/f1000research.190182.r428397

**Published:** 2025-11-17

**Authors:** Dr Sunita Saldanha

**Affiliations:** 1 Yenepoya School of Allied Healthcare Professions, Manglore, Karnataka, India

I have gone through the revised article. However, the study’s scientific outcome is questionable   because

 The Study findings are only limited to the application of WISN for the calculation of workload, which is well-established, globally accepted fact & it is WHO-endorsed tool

 The question remained unanswered: What is the rationale of selecting WISN for automated laboratory environments?

 Or how HR can utilise this finding for recruitment? Workload Balancing? Budgeting?

 Is this method preferable over any other method?

 With the integration of Automation, the WISN 's Standard activity metrics (time/Task) can be tailored for laboratories. 

 Why WISN is preferred over ratio-based, norm-reference, or qualitative workload assessment or any other accepted methods?

 By acknowledging and addressing these points, the work can serve as an evolving benchmark for human resource planning and management in contemporary clinical laboratories.

 I recommend substantial revision to address these concerns and enhance the impact and scientific value of the study.

Is the work clearly and accurately presented and does it cite the current literature?

Yes

If applicable, is the statistical analysis and its interpretation appropriate?

Partly

Are all the source data underlying the results available to ensure full reproducibility?

Yes

Is the study design appropriate and is the work technically sound?

Partly

Are the conclusions drawn adequately supported by the results?

Partly

Are sufficient details of methods and analysis provided to allow replication by others?

Partly

Reviewer Expertise:

Hospital administration , Quality & patient Safety

I confirm that I have read this submission and believe that I have an appropriate level of expertise to confirm that it is of an acceptable scientific standard, however I have significant reservations, as outlined above.

# Response

**Published:** 2025-12-10

**Authors:** DR ASHA PATIL

**Affiliations:** Medical Laboratory Technology, Manipal Academy of Higher Education, Manipal, Karnataka, India

Thank you for your thoughtful and detailed feedback. We truly appreciate the opportunity to strengthen our manuscript and have made substantial revisions to address your concerns.

**Why WISN for Automated Laboratories- **We have addressed in the **Introduction** - Automation improves efficiency, it does not replace the need for skilled professionals for pre-analytical, analytical oversight, troubleshooting, and post-analytical tasks. WISN was chosen because it is a WHO-endorsed, evidence-based tool that uses real workload data and can adapt to partially automated environments.

**How WISN Was Adapted for Automation-**In the **Methods** section- we have explained that activity standards (time per task) were customized to reflect automation-related changes in task duration and complexity. This ensures that WISN remains relevant in modern laboratories.

**Practical Value for HR Managers-** In **t**he **Discussion** we have ****expanded to show how WISN ratios can guide recruitment planning, workload balancing, and budgeting decisions. These additions demonstrate that WISN is not just a calculation tool—it’s a strategic resource for workforce planning.

**Why WISN Over Other Methods- **We have added a comparison highlighting WISN’s advantages over ratio-based, norm-referenced, and qualitative approaches. WISN offers transparency, reproducibility, and flexibility—qualities that other methods lack.

**Positioning WISN as a Benchmark- **The revised text emphasizes that WISN’s adaptability and evidence-based approach make it a benchmark for workforce planning in evolving healthcare environments where technology and human expertise must coexist.

# Reviewer report: Reviewer response for version 2

**DOI:** 10.5256/f1000research.190182.r428396

**Published:** 2025-11-12

**Authors:** Nasar Yousuf Alwahaibi

**Affiliations:** 1 Biomedical Science Department, College of Medicine and Health Sciences, Sultan Qaboos University, Muscat, Oman

It is much better now, however, the authors did not address the below important issue: 

 A clear sample size calculation is missing. This is essential to assess whether the study was adequately powered to draw meaningful conclusions.

Is the work clearly and accurately presented and does it cite the current literature?

Partly

If applicable, is the statistical analysis and its interpretation appropriate?

Partly

Are all the source data underlying the results available to ensure full reproducibility?

Partly

Is the study design appropriate and is the work technically sound?

No

Are the conclusions drawn adequately supported by the results?

Partly

Are sufficient details of methods and analysis provided to allow replication by others?

No

Reviewer Expertise:

Histopathology

I confirm that I have read this submission and believe that I have an appropriate level of expertise to confirm that it is of an acceptable scientific standard, however I have significant reservations, as outlined above.

# Response

**Published:** 2025-11-16

**Authors:** DR ASHA PATIL

**Affiliations:** Medical Laboratory Technology, Manipal Academy of Higher Education, Manipal, Karnataka, India

Thank you for your comment regarding sample size.

 We have addressed this in the Methods section: the study involved complete enumeration (n=33) of the available laboratory workforce, so an a priori sample-size calculation was not required (all 33 technical staff were included). Similarly, a power calculation was not applicable because this was a census study; conventional formulas for sample size and power assume sampling, which was not undertaken here.

 The focus of the study was a descriptive assessment of workload and staffing requirements for the entire group, rather than hypothesis testing, which is why formal sample size and power calculations do not apply to this design.

# Reviewer report: Reviewer response for version 1

**DOI:** 10.5256/f1000research.167552.r400545

**Published:** 2025-09-19

**Authors:** Nasar Yousuf Alwahaibi

**Affiliations:** 1 Biomedical Science Department, College of Medicine and Health Sciences, Sultan Qaboos University, Muscat, Oman

As a Q1 journal committed to maintaining high scientific and methodological standards, I recommend not approving this manuscript. The study contains substantial flaws in both the Methods and Results sections, which undermine the validity and reliability of the findings. These issues must be thoroughly addressed before the manuscript can be reconsidered for indexing.

**Strengths:**

- The topic of optimizing clinical laboratory staffing using the WISN method is relevant and potentially valuable for healthcare workforce planning.
- The authors have attempted to address a practical issue with implications for laboratory efficiency and quality of care.

**Limitation:**

- The title is overly long and includes abbreviations, which should be avoided.
- The abstract requires complete rewriting to better reflect the structure of a scientific summary. The current methods section of the abstract incorrectly states the study aim rather than describing the methodology applied.
- The manuscript inconsistently uses the term "lab/s." The full term “clinical laboratory/laboratories” should be used throughout for clarity and professionalism.
- A clear sample size calculation is missing. This is essential to assess whether the study was adequately powered to draw meaningful conclusions.
- Table 1 title should be revised to be more specific, and it would be helpful to include the number of tests performed per year.
- Results are currently presented in narrative form; they would benefit from tabular and/or graphical representation for clarity and better interpretation.
- The methodology lacks detail and structure.
- The statistical analysis is incorrectly placed in the Results section. It should be described under the Methods section
- The data were collected during the COVID-19 pandemic, which could have significantly influenced staffing patterns and workload. However, the manuscript does not mention or discuss the potential impact of the pandemic on the findings.
- The issue of financial allowances or compensations for overwork is raised but not adequately addressed or analyzed. This aspect needs more clarity and data support.

 

 In summary, the manuscript requires substantial revisions to meet the scientific and methodological standards expected of a Q1 journal.

Is the work clearly and accurately presented and does it cite the current literature?

Partly

If applicable, is the statistical analysis and its interpretation appropriate?

Partly

Are all the source data underlying the results available to ensure full reproducibility?

Partly

Is the study design appropriate and is the work technically sound?

No

Are the conclusions drawn adequately supported by the results?

Partly

Are sufficient details of methods and analysis provided to allow replication by others?

No

Reviewer Expertise:

Histopathology

I confirm that I have read this submission and believe that I have an appropriate level of expertise to state that I do not consider it to be of an acceptable scientific standard, for reasons outlined above.

# Response

**Published:** 2025-10-23

**Authors:** DR ASHA PATIL

**Affiliations:** Medical Laboratory Technology, Manipal Academy of Higher Education, Manipal, Karnataka, India

We sincerely thank the reviewer for their constructive feedback and insightful suggestions.

**Response to Reviewer Comments**

**Title**

  We agree and have revised the title to: Workload Assessment of Technical Staff in a Clinical Laboratory in South India.

**Abstract**

 The abstract has been revised in a structured format (Background, Methods, Results, Conclusion). In the Methods section, we now provide a concise description of the WISN methodology, data sources (laboratory statistics over two years), and statistical tests applied, instead of restating the study aim.

**Terminology**

 We have carefully revised the manuscript to replace “lab”/“labs” with “clinical laboratory/laboratories” consistently.

**Table 1**

 Table 1 has been revised and retitled:

 Workload components, standard workload, annual number of tests, and calculated staff requirements.

**Results Presentation**

 The Results section has been revised to improve clarity and interpretation by including tabular presentations. Specifically, one table summarizes the workload components, standard workloads, and calculated staff requirements (Table 1), and a second table presents actual versus WISN-based staffing with corresponding WISN ratios and workload pressure (Table 3). These tables provide a clear and comprehensive view of staff requirements and workload distribution across clinical laboratory sections.

**Methods vs. Results**

 We agree. The description of the Shapiro–Wilk test and Kruskal–Wallis test has been moved from the Results to the Methods section. Only the test outcomes are retained in the Results.

**COVID-19 Impact**

  We thank the reviewer for this important point. We have now addressed this in the Limitations section:

 The data were collected during the COVID-19 pandemic, which may have influenced staffing availability and workload patterns (e.g., absenteeism, emergency testing demands). This context may partly explain the higher workload pressure observed.

**Financial Allowances / Compensation**

 We acknowledge this concern. We have clarified in the Discussion that WISN is a workforce planning tool and does not account for financial compensation.

 We have added the following statement under - Future scope of the study: ****Financial allowances and compensation are important considerations in managing overworked staff, however, the WISN tool does not directly assess these aspects. Future studies incorporating economic assessments and cost benefits analysis alongside WISN analysis would provide a more comprehensive understanding of the workforce planning and staff motivation.

**Overall Framing / Technical Soundness**

  We respectfully clarify that our study was designed as a workload assessment using the WHO-developed WISN methodology. While it is not a hypothesis-driven clinical trial, it systematically applies an internationally recognized tool to evaluate workforce adequacy in a real-world setting. We have strengthened the Methods section to provide clearer structure and detail and revised the Discussion to focus specifically on workload assessment (rather than utilization), ensuring the findings are appropriately contextualized.

**Conclusion**

 We have addressed the reviewer’s suggestions, including restructuring the abstract, refining the Methods and Results sections, revising terminology, updating tables and figures, and expanding the discussion of limitations. We believe these revisions have substantially improved the scientific and methodological clarity of the manuscript.

 We appreciate the reviewer’s insightful comments, which have significantly improved the clarity, focus, and overall quality of the manuscript.

# Reviewer report: Reviewer response for version 1

**DOI:** 10.5256/f1000research.167552.r363041

**Published:** 2025-02-11

**Authors:** Dr Sunita Saldanha

**Affiliations:** 1 Yenepoya School of Allied Healthcare Professions, Manglore, Karnataka, India

Dr. Asha Patil and co-authors have addressed a crucial and important concern regarding the health workforce, which presents significant challenges for Health Service Managers. The study focuses on the workload of technical staff in the flow cytometry section, which is a valuable contribution to understanding staff management in this domain.

 However, there are a few issues that need attention. The title and methods section mention staff utilization, but this aspect is not sufficiently addressed in the study. Since the research focuses on workload rather than utilization, it would be more appropriate to revise the title to reflect the study's actual focus.

 Additionally, the study is limited to one hospital in South India, and it does not include data from other hospitals. Expanding the scope or acknowledging this limitation would enhance the generalizability of the findings.

 Minor corrections are also recommended regarding the use of the short form "WISN" (Workload Indicators of Staffing Needs) throughout the article to ensure consistency.

 Further clarification on the calculation of service standards and allowance factors would be beneficial for better understanding. Specifically, the first WISN step, which determines staffing priorities, should emphasize the inclusion of staff based on the work's importance, rather than detailing which staff works in specific sections. This will help clarify the intent of the methodology.

 In the discussion and conclusion sections, particularly on Page No. 7, there is a need for better clarity. Some paragraphs appear incomplete, and the discussion should focus more on workload assessment and its importance, rather than on utilization, as the current analysis does not provide insights into manpower utilization.

 These revisions will contribute to the overall clarity and precision of the study.

Is the work clearly and accurately presented and does it cite the current literature?

Yes

If applicable, is the statistical analysis and its interpretation appropriate?

Partly

Are all the source data underlying the results available to ensure full reproducibility?

Yes

Is the study design appropriate and is the work technically sound?

Partly

Are the conclusions drawn adequately supported by the results?

Partly

Are sufficient details of methods and analysis provided to allow replication by others?

Partly

Reviewer Expertise:

Hospital administration , Quality & patient Safety

I confirm that I have read this submission and believe that I have an appropriate level of expertise to confirm that it is of an acceptable scientific standard, however I have significant reservations, as outlined above.

# Response

**Published:** 2025-10-23

**Authors:** DR ASHA PATIL

**Affiliations:** Medical Laboratory Technology, Manipal Academy of Higher Education, Manipal, Karnataka, India

We sincerely thank the reviewer for their constructive feedback and insightful suggestions.

 The title and manuscript have been revised to focus exclusively on *workload assessment* rather than *staff utilization*, ensuring alignment with the study’s actual objectives.

 Explanations of the WISN methodology, including service standards and allowance factor calculations, have been added for clarity.

 The discussion and conclusion sections revised to emphasize workload findings, with reduced focus on utilization.

 A statement acknowledging the limitation of data being drawn from a single hospital has also been included to address generalizability concerns.

 Furthermore, WISN terminology and abbreviations have been standardized throughout the manuscript for consistency and accuracy.

 We appreciate the reviewer’s insightful comments, which have significantly improved the clarity, focus, and overall quality of the manuscript.
